# The evaluation of an evidence-based model of feedback implemented on an undergraduate dental clinical learning environment

**DOI:** 10.1186/s12909-022-03630-1

**Published:** 2022-08-01

**Authors:** Siobhan Davis, Brett Duane, Andrew Loxley, Duana Quigley

**Affiliations:** 1 Dublin Dental University Hospital, Lincoln Place, Dublin 2, Ireland; 2 School of Education, Trinity College Dublin, Dublin, Ireland; 3 Department of Clinical Speech and Language Studies, Trinity College Dublin, Dublin, Ireland

**Keywords:** Feedback, Assessment, Dental education, Model of feedback, Clinical learning environment

## Abstract

**Objectives:**

Dental graduates must graduate with high levels of clinical skills. Education in the clinical environment needs to be more than didactic supervision of practice by clinical teachers. Appropriate feedback in this context, is therefore critical to the development of student competence and confidence. This study was conducted to enhance and develop the assessment and feedback processes during clinical sessions in a Dental University Hospital in an effort to contribute to the development of students’ self-assessment skills, reflective ability and clinical competence.

**Methods:**

A new evidence-based model of feedback was introduced between clinical teachers and dental students. The implementation of this model was evaluated by students through a survey and focus groups. Descriptive and inferential statistics were applied to the quantitative data, while thematic analysis applied to the qualitative data.

**Results:**

Findings from the survey indicated that students perceived the new model of feedback to be a positive addition to their learning experiences. The majority indicated a preference to continue using it. Quantitative analysis also demonstrated that students placed a high value on the feedback they received through the new model and associated it with improved individual performance. Five themes generated from the qualitative analysis echoed the perception that the model of feedback enhanced learning opportunities, especially when it was focused on individual performance and incorporated peer feedback. Students’ preferences in relation to feedback processes were also gleamed from quantitative and qualitative analyses, that is, provision of positive and constructive feedback, both in dialogue and in written formats, delivered during and after each clinical session and addressing their individual competency learning goals for the future. Some challenges to be addressed were also identified (e.g., time constraints, inter-personal issues, and non-conducive environments).

**Conclusions:**

Feedback is central to learning and remains a complex and challenging area. By adopting effective and evidence-based feedback practices through the introduction of a feedback model, students can be supported in regulating their own learning in the clinical learning environment.

**Supplementary Information:**

The online version contains supplementary material available at 10.1186/s12909-022-03630-1.

## Introduction

Central to the supervision of dental students is the provision and receipt of feedback. Feedback typically has three components which include the identification of clear goals, an indication of the students’ performance against these goals, and guidelines on how to improve in future work [1]. Feedback is fundamental to facilitating students’ development as independent learners, who have the ability to monitor, evaluate and regulate their own learning [2]. The development of self-assessment abilities is desirable to encourage professionalism, life-long learning, and competency in the dental graduate [3]. Effective feedback can greatly enhance the student experience. Feedback is also important in ensuring a quality educational experience for students and enhancing the engagement of students in their course of study [4].

However, feedback can be difficult for both clinical teachers and students and the clinical learning environment is universally deemed challenging [5]. Several barriers to effective feedback from the clinical teachers’ perspective include, time constraints, high work demands, difficulty engaging multiple levels of learners and a clinical environment not comfortable for teaching [6] Studies have also documented challenges in relation to student seeking feedback practices which include defensiveness, non-engagement, emotional distress, and limited understanding of the feedback process [4, 7, 8]. Despite the challenges on both sides, the importance of feedback is paramount to ensure mistakes are avoided, excellence is reinforced, and continuous work towards expected standards is being achieved. Therefore, there is a need to educate and support clinical teachers and students alike in feedback practices and enhancement of their feedback literacy skills must not be overlooked [4].

Feedback can be given in a structured, highly regimented way or in a more unstructured ad-hoc manner [9]. Traditional perspectives tend to construe feedback as monologue of information transmission, which is at odds with more contemporary views of effective feedback being a dialogic, dynamic, interactive, and two-way process [8, 10]. This has been compared to a shift in construing feedback from a ‘product’ to a ‘process’ [11] and from ‘disclosure’ (i.e., students hearing about the quality of their work) to ‘visibility’ (i.e., students understanding the reasons for quality) [12]. Carless et al. [13] describes ‘sustainable feedback’ which is founded on dialogue, self-evaluation, and goal-setting that facilitates life-long learning. Some models of feedback have been proposed that include structured sequences in an attempt to enhance students’ and teachers’ knowledge of what is expected of them during a feedback session and promote more evidence-based feedback processes and practices [13]. For example, Pendleton’s Model (i.e., you ask what went well?; you tell what went well; you ask what could be improved?; you tell what could be improved) [14], Sandwich model (i.e., praise, constructive criticism, praise) [15], EEC (i.e., example, effect, change/congratulate) [16] and the Chicago model (i.e., review aims, interim feedback of a positive nature, ask learner to give self-appraisal, give feedback focusing on behaviour, suggest strategies for learners to improve their performance) [17].

Based on a review of the literature, a good model of feedback should incorporate and promote several core evidence-based feedback practices to ensure its effectiveness. It should promote reflecting in learning [18] and the development of self-assessment skills such as understanding standards and gaining experiences in making judgments [10, 19]. It should incorporate the development of self-regulating skills, that is, the ability to support the student to regulate their thinking, emotions, motivation, and behaviours during learning [12, 20]. It should encourage engagement in dialogic and interactive discussions to help the learner make sense of the learning [19]. The delivery of high quality information to students about their learning that is clear, positive, individualised, jargon-free and future-oriented should be present [21]. It should support positive motivational beliefs and self-esteem [21]. Furthermore, it should provide opportunities to close the gap between current and desired performances through goal setting and action plans [10, 21] and should focusing on a feed forward [22] longitudinal development of learning. The former evidence-based practices are largely complementary in nature to enable a model for effective feedback for clinical teachers and students to be developed.

There are three key factors providing the rationale for this study. First, the former feedback models position teachers as the drivers of feedback. This may ignore student agency and neglect the importance of student engagement. Thus, there is also a need for a model of feedback (MOF) that positions the learners as having a key role in driving learning and draws on ideas of sustainable assessment [4] Second, without a defined and consistent model of feedback to implement that incorporates educator and student priorities, feedback provided to students may be unstructured, leading to several different methods being employed by different clinical teachers. This may result in numerous inconsistencies, subjectivity, lack of transparency, and diverse expectations. A new MOF can help address these various inconsistencies and increase clarity of expectations. Third, in the dental clinical environment, the critical aspect of patient safety is paramount and the amalgamation of a preparatory step to the model of feedback in advance of a student’s performing a clinical intervention or activity is deemed necessary to be incorporated into a model of feedback to minimise risk and ensure patient safety.

### Aim

The aim of this study was to develop, implement and evaluate the introduction of a new evidence-based model of feedback in a Dental University Hospital.

## Materials and methods

This study was designed as a mixed method study. The study protocol and the two phases of evaluation completed are outlined below. The study protocol was reviewed and approved by the Research Ethics Committee of School of Dental Science, Dublin, Ireland(DSREC2016-10).

### Description of the intervention

The new model of feedback (MOF) introduced in this study was adapted from Nicol and MacFarlane-Dick [21] and incorporated many of the above recommended feedback practices (see Table 1 for a full description of the MOF). A structured MOF consisting of six key steps (Table 1) was developed by the first author based on the evidence-based practices outlined earlier. The model was designed specifically to suit the dental learning environment, with the addition of a preparatory step which was necessary to ensure patient safety. This MOF had been previously piloted with a group of students (*n* = 8) earlier in the academic year. Implementation of the MOF involved a number of steps. First, a presentation was delivered to clinical teachers introducing the MOF. The clinical teachers were requested to keep fidelity to the six steps of the MOF for the duration of the study (i.e. four weeks), thus ensuring calibration in the use of the MOF. Second, all clinical teachers were emailed a link to an assessment questionnaire that evaluated their understanding of effective feedback principles and the new MOF. All clinical teachers completed and passed the assessment (achieving a score of 90% or higher) prior to the commencement of the study period. Third, a feedback process between teachers and students using the MOF was implemented through a verbal, face-to-face, synchronous exchange. Fourth, a checklist with instructions for clinical teachers was provided for the study period to ensure all points of the model were implemented during the feedback exchange about the student’s clinical session.

**Table 1:** Outline of new Model of feedback introduced

1. Learning outcomes are highlighted at beginning of the clinical session verbally –student must know what they are setting out to achieve at the start of the clinical session.
2. Example of good work is shared with the student (e.g., refer to a textbook or online material) prior to the session so that student knows what s/he is striving to achieve or to model good practice.
3. Feedback to the student incorporates a reflective component, for example, “How do you feel that went? What would you do differently next time?”
4. Clinical teacher determines if any issues arose for the student over the session based on expected performance/standards that could be improved for the next clinical session.
5. Clinical teacher enters a dialogue with the student, highlighting what went well, any issues which occurred over the clinical session and how they could be addressed. The student is advised to keep a written record to guide their learning before the next session.
6. Students are asked how they are progressing with their learning goals, any areas of concern or need for clarification.

### Participants

This study was conducted at Dublin Dental University Hospital, Dublin, Ireland. The undergraduate dental programme is a five-year programme leading to the award of Bachelor of Dental Science. Students are taught by a combination of problem-based learning (PBL), didactic lectures, clinical skills laboratory, and chairside clinical teaching under supervision. The learning is monitored, supported, and evaluated using formative and summative assessment.

Undergraduate students treat patients in the final three years of the course. During the timeframe of the study, there were a total of 77 undergraduate students in the 3rd and 4th Years, 45 in the former and 32 in the latter. Each year is divided into six modules consisting of 6–8 students. Participants were informed that the study was restricted to the restorative clinics, namely, Basic Dental Care, Integrated Patient Care and Advanced Restorative Care clinics in the 3rd and 4th year dental undergraduate students for a 4-week period. Prior to consenting to participate in the study, all potential participants were informed of the nature of the study through a participant information leaflet and an oral presentation. They were invited to ask questions and if willing to be a participant, they completed a written consent form after an appropriate period of “cooling off”.

Forty-four students consented to participate, 35 were from the 3rd year cohort and nine from the 4th year cohort. All student participants were provided with information on the new MOF that was being introduced for a 4-week period via an oral presentation by the first author entitled “The implementation of an evidenced-based model of feedback for undergraduate dental students on clinical sessions”.

### Evaluation

Student participants were involved in two phases of evaluation of the MOF: (i) evaluation via an online survey; and (ii) evaluation via focus groups.

#### Phase 1: evaluation of the new MOF via online survey

 After the 4-week study intervention period had been completed, participants received an email link to an anonymous online survey to gather their perspectives about the new MOF (see Additional file 1: Appendix 1). The survey contained 17 questions, that included Likert scales, multiple choice questions, and free field comments. To preserve anonymity, the questionnaire did not collect any personal data or any IP addresses. The analysis of the quantitative data was completed through descriptive and inferential statistics using the Statistical Package for the Social Sciences (SPSS), Version 24.0 (IBM Corp., Armonk, NY) and Excel, Version 19 (Microsoft Corp., Washington U.S.).

#### Phase 2: evaluation of the new MOF via focus group

**Fig. 1:**
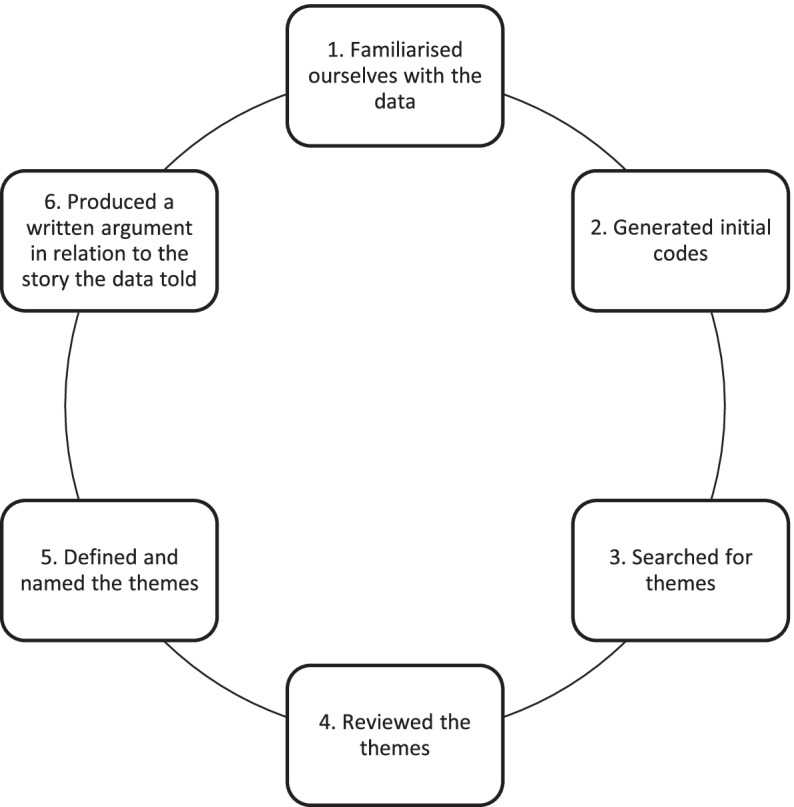
Six phases of thematic analysis completed, as described by Braun and Clarke

The 44 participating students, following completion of the online survey, were then invited to participate in a 20-minute audio-recorded focus group to enable further rigorous evaluation of the MOF. The focus group questions (Additional file 1: Appendix 2) were based on the post-evaluation survey. These questions were piloted and minor modifications were made to ensure efficacy of the focus groups. A total of six focus groups were facilitated with eight students in four of the focus groups and six students in two focus groups. All focus groups were conducted by BD and AL, who were not involved in the clinical training of the participants and were facilitated based on published focus group interviews methodology [23]. At the start of the focus group, the moderators reminded the participants of the principles of a focus group and reassured them that there were no correct or incorrect responses, and that confidentiality and anonymity would be upheld. A neutral and impartial view was taken by the moderators. All participants were encouraged to speak, and the moderators made every effort to ensure that one or two people did not dominate the discussion. Anonymity was protected by transcribing the data and using a coding key (e.g., Group A participant 1, Group C participant 2 etc.), thus ensuring that no participant was identifiable. The focus groups were transcribed anonymously by a third party. Thematic analysis was then applied to the data following Braun and Clarke’s six phases of thematic analysis as outlined in Fig. 1 [24].

Once the researchers had familiarised themselves with the data and generated initial codes, all codes were gathered, and duplicate codes were removed. We searched for themes by identifying overlapping or close codes and associated clusters of codes. Consequently, five themes were generated to capture patterns of meaning related to the research aims that were within the data set. The themes were reviewed, defined and named, until a consensus was reached by all authors.

## Results

### Phase 1: results of evaluation of new MOF via online survey

**Fig. 2:**
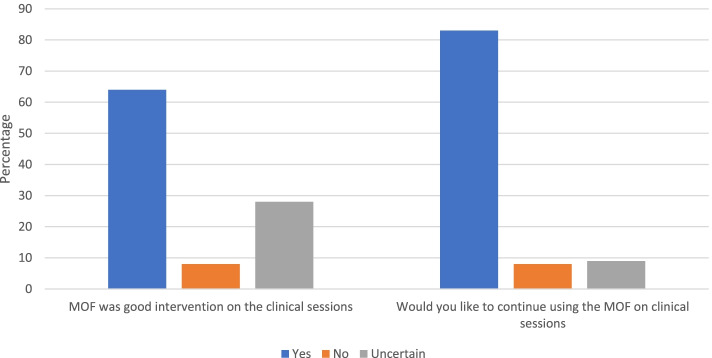
Results from the quantitative data in evaluation of the MOF

The online survey was completed by 44 students out of a total of 77 students (57%) in this 3rd and 4th year student group. Over three-fifths (64%) indicated that the MOF was a good intervention (Fig. 2). The data also showed that the majority (83%) expressed a preference to continue using this MOF during clinical sessions (Fig. 2). A minority of participants (5%) suggested there were points in the MOF that they had difficulty with.

In addition, almost all (98%) of students surveyed said it was important for them to receive regular feedback on their work. Over three quarter of students (77%) linked an enhancement in their performance on clinical sessions to feedback received. 39% of students felt they were receiving sufficient feedback on their work at the dental school, with 27% reporting they were not. Challenges limiting their ability to receive feedback included time constraints (66%), difficulty engaging with some of the clinical teachers/academic staff (36%), and an environment not conducive to feedback-seeking practices (30%). 61% of students surveyed felt able to discuss the feedback they received with their clinical teacher in the clinical environment. 61% of students surveyed would prefer to receive feedback in both written and oral forms. Aspects of feedback found to be of value included feedback from a clinical teacher familiar with their work (73%), knowing how to improve on their work/grade (86%) and an indication of where they went wrong (84%). 84% of students also indicated they would like the feedback to incorporate a comment in addition to their grade. When asked about the current feedback processes at the dental school, 36% said they were good, 41% just satisfactory and 21% unsatisfactory.

Finally, when asked if they would like to see a change in future feedback processes having used the new model of feedback implemented in this study, over two-thirds (68%) of students surveyed said yes, with 14% answering no to this question.

### Phase 2: results of evaluation of new MOF via focus group

44 students (57% out of a total of 77 students) from two years consented to participate in a 20-minute audio-recorded focus group to enable further rigorous evaluation of the model of feedback. Six focus groups were facilitated, with six to eight participants per focus group. Based on the thematic analysis of the data that was collected, five themes were generated and these themes permeated the data.

#### Theme 1: MOF enhanced learning opportunities

Students made comparisons between the new streamlined MOF amongst all clinical teachers and previous ad-hoc feedback experiences.

*“the best way to describe it was non-standardised; it really depended on the teacher”* (Participant A3)

Several participants suggested that using the MOF led to a more positive experience during clinical sessions, such as contributed to learning opportunities. It was important for students to have feedback from a clinical teacher who gave them accurate information about their performance and could also be used to benchmark their performance in relation to the group under supervision and the expected standards for their stage in the programme.

*“I think we all want to do a good job so that feedback is very valuable in terms of assessing our own performance. It doesn’t always tell you where you need to change or what you need to work at but ****that ****feedback is extremely valuable for improvements to be made”* (Participant A4)

Students described an improvement in the quality of feedback they received.

“*One of our teachers wrote little comments… it was actually really helpful because afterwards you could look back in your grades”* (Participant E5)

In virtually all of the focus groups the students reported an association between feedback on their learning in the clinical environment and enhanced education.

*“It’s amazing how much I learn on the clinics. The MOF helped show how much learning we do every day on the clinical sessions”* (Participant D6)

In some situations, the MOF contributed to short discussion groups to support and supplement learning. Some students described how the dialogue inherent in the MOF supported a deeper level of learning by helping them to draw on learning from previous sessions, provide more detailed and nuanced information and link it with current experiences.

*They give you more information on where you went wrong, what you can improve on*. (Participant E5)

#### Theme 2: Preference for feedback that is future orientated

Students’ appraisal of the MOF indicated that they rated positively feedback that showed them where and what aspects went well, so that these can be replicated, repeated, and improved on in future clinical sessions.

*“I found that while using the MOF on my interactions with the clinical teacher I was able to apply the knowledge and skills I learned from the previous clinical session to improve my performance”* Participant B2.

*“Most people in the class are aiming for high performance so you want to continually improve and not be performing at the bottom of the class”* Participant D4

This contrasts with previous experiences shared by students when there was a lack of external validation from the clinical teacher, which was not perceived as satisfactory.

*“If you’re not actually told, … you think everything was fine and you get a bad grade and you might not even realise it.”* (Participant E2)

*“With the feedback I had been given it didn’t really tell me anything about what I have to improve”*. (Participant D3)

Students also expressed a preference for both formative and summative assessment on the clinical sessions to help them improve future performance.

*“I think the comment beside the grade is a good idea because it’s confidential … and its constructive…and eliminates the issue of time because there no need for you to go up to the clinical teacher after every session, they can always upload that comment afterwards, so you are always getting feedback”* (Participant D5*)*

#### Theme 3: Preference for feedback that is focused on individual competency learning goals

Participants across all focus groups rated positively all feedback through the MOF that was performance-related feedback, pertaining to patient care, treatment plans, technical ability/skill, and time management, as they found this beneficial in relation to their learning on the clinical sessions. Students wanted to know how they can improve aspects of treatment and commended feedforward components related to their learning for future clinical sessions. They suggested providing additional feedback and information about interventions for skills enhancement which could be provided to facilitate the translating of knowledge into improving their individual practice.

*“…if you’re shown and discuss the steps initially and you do them right and work on them… and guided through that, you will get quicker a lot faster”* (Participant D3).

“*Are you doing it right, are you putting it in slow enough or are … there’s subtle nuances”. …I think in dentistry…we don’t know what it is we need to learn… until you encounter a situation”* (Participant E2)

Most agreed that learning outcomes should be tailored to students *“your own individual needs”* (Participant A2). However, of note, students believed that if they critiqued their own performance, in a feedback dialogue, that they would be penalised on the grade.

*“…they think if they’re going to get a bad grade if they identify…problems with their work… and that’s going to reflect on your grade”* (Participant E3)

#### Theme 4: Preference for more time for feedback

The ability of the MOF to secure dedicated time for feedback on the clinical sessions emerged as a major theme across all of the focus groups.

*“there’s no time constraint…” (*Participant D3).

This was typically highlighted by making comparisons with previous experiences when feedback would have been rushed or there was a time lag between the clinical session and receiving the feedback.

*“You’re meant to have the patient out of the chair by the time, your notes written up by this time” …”in terms of incorporating feedback unless you have a specific time for it… I don’t think… it gets done”* (Participant D3).

*“…by the end of the week you (have) had so many sessions you don’t remember anything”* (Participant B2)

#### Theme 5: Benefits of peer feedback

The benefits of the MOF facilitating peer feedback also emerged as a major theme across all focus groups. Students reported that peer experience, peer-learning and peer feedback were important opportunities in the clinical learning environment.

*“You’re discussing that over and back because you’re learning from other people’s experiences”* (Participant B3).

*“I share my mistakes with my friends… so they wouldn’t repeat what I did…it’s sort of helps me not to do the same mistake again… you saw tips and tricks; I would usually, usually share…”* (Participant B5).

Participants understood the importance of having colleagues with whom they could share reviews of their performance with and have access to networks that they could seek improvement through.

*“I like to compare myself to other people with a similar experience or similar level as myself. That way I know how I am performing in relation to my group.” (*Participant F1)

## Discussion

This study aimed to evaluate the introduction of a new six-step MOF through a mixed methods study. Based on the quantitative and qualitative data analyses, a number of key findings were generated.

First, this study highlights that students value their learning and place a high value on feedback. Almost all of the students surveyed (98%) reported that they believed it was important to receive regular feedback on their work. Wiggins [25] agreed that effective feedback should be timely, and dependent on the context of the learning and the needs of the learner [2, 26, 27]. To be effective it must be given to the student while it still matters to them on work in progress and also in time for them to use it to feed-forward into their next assignment or task [28] This MOF provides a framework for provision of regular effective and evidence-based feedback.

The literature supports the importance of clinical teacher feedback and engaging with students’ perceptions and use of feedback [29] While 64% of participants rated the new MOF as a good intervention and 83% expressed a preference to continue using it, some student dissatisfaction was also identified with some students of the opinion that they did not receive enough feedback. Student dissatisfaction with feedback processes has been reported elsewhere too [30]. As integrated in this MOF, effective feedback should encourage sufficient interaction and dialogue with teachers and peers as a way to make sense of the learning [26, 31, 32]. The literature would also suggest that students need to be engaged in and with this two-way process and the importance of interactive and dynamic feedback processes to ensure high quality feedback experiences [19, 29]. The multi-dimensional performances which are present in assessment in higher education mean that the feedback must match this level of complexity, and this may pose challenges to developing quality supervisory relationships and safeguarding time and support to develop feedback literacy skills of students and supervisors [4, 33] Different modes of delivering effective professional development for clinical teachers may promote and enhance understanding of feedback processes [28, 34].

Second, time constraints in delivering this feedback was identified as the most limiting issue in the student’s ability to receive the feedback they need. This has also been observed as a main barrier by others [22]. Our results mirrored those of Spencer [35] who reported time barriers such as other work demands, difficulties in engaging multi-levels of learners, uncomfortable physical environment and lack of incentives. The issue of students and clinical teachers having differing finish times on the clinical sessions is recorded as posing a barrier to feedback on clinical sessions. With regards to timing, it is suggested that immediate feedback is possibly the most effective in the context of clinical skills acquisition and training [22]. Consideration needs to be given to providing an appropriate time for feedback possibly in dedicated scheduled slots and it is recommended that this be included in future iterations of the MOF.

Third, an additional barrier was the reluctance of many students to approach staff to seek feedback, as they were worried this might be reflected negatively in their grade at the end of the clinical session. This is at odds with principles of good feedback literacy skills, whereby students are active agents in a two-way feedback process [10]. As integrated into the steps of the MOF, good feedback practices should demystify the assessment process by providing explicate guidance to clinical supervisors and students in relation to assessment criteria and what quality is and modelling good practice [36, 37]. Students can benefit from checking their grades and feedback regularly in order to monitor their own performance against established standards and develop self-regulation in the process. Good feedback practice is frequently described as anything that might strengthen the student’s capacity to self-regulate their own performance [21]. Self-regulation is the ability of the student to regulate their thinking, motivation and behaviours during learning [20]. It would appear that this aspect of the MOF may require further attention from clinical teachers and students, such as additional and more details focused on developing problem-solving skills, promoting critical thinking and self-directed learning to help enhance feedback literacy skills [38]. Engaging in constructive dialogue can be a challenge and is an important factor in successful feedback with trust being central to the process [19, 39]. Student involvement in the process and understanding of the learning process is central to the development of evidence-based feedback practices and may require further explicit instruction, role play and practice [40]. Educators should support students in realising this central role and support them with confidence in meeting this role [41].

Fourth, it was highlighted that the amount of feedback received should be manageable rather than an endless task to the providers of feedback and also to the students [28, 33]. Getting too much feedback can result in an inability to discern the important feedback from the routine feedback. Balanced against this is the fact that feedback needs to be effective, and in order to achieve this, it must have sufficient detail, be given to the student while it still matters to them on work in progress [21] and also in time for them to use it to feed-forward into their next assignment or task [22]. When a single word for feedback is used (e.g., ‘productivity’ or ‘technical skill’), the feedback lacks direction and contains no signposting for future learning. In higher education, the central argument is that formative assessment (assessment that is specifically designed to generate feedback for future learning) should be utilized to empower students as self-regulated learners [21]. Therefore, perhaps more guidance on the volume of feedback to provide should be included in future iterations of the MOF (e.g., feedback should be of sufficient detail that the student is aware of the current performance compared to expected performance and has a clear action plan of how to improve). To assist with the volume of feedback, it may be provided in both oral and written forms, and it can be formal or informal, individual or group, specific or generic, self or peer [33]. Written feedback is recorded and may be reflected on later by the student and may as such promote reflection on learning.

Fifth, the value of peer feedback was highlighted in this study. Although the MOF was designed as a framework for clinical teachers and students, its principles could easily be applied to peer feedback. Others have demonstrated educational gains through peer feedback systems [42]. Peers may be able to provide effective additional feedback to the learner, again satisfying the students’ request for increased volumes of feedback. Learners can also learn more themselves from the act of giving feedback; the greater cognitive gain is usually from the peer tutor [43, 44]. Dialogue about their performance and knowledge with peers gives rise to opportunities about what they are learning and how this links to performance and knowledge. Peer based feedback does not come from a clinical teacher, who often has evaluative power over the learner, which can impact learning greatly. Students may not want to reveal a lack of knowledge and performance weaknesses to the clinical teacher [4]. The students valued the intervention and felt supported in the process of feedback in the clinical learning environment.

A report and five broad recommendations (Table 2) were made in a presentation to the academic staff based on the data collected in this study. The findings of this study helped with the development of an eLearning module in Feedback in the Clinical Learning Environment for dental and other interdisciplinary healthcare education programmes. Moreover, a similar module to support student literacy in feedback is planned for development in the future.

**Table 2:** Recommendations from the study on the feedback

	Recommendations from the study on feedback.
1	Feedback is important to students, should continue to be provided and needs to be improved within DDUH.
2	The DDUH should consider how it may alleviate the time pressures associated with giving appropriate feedback. Feedback should be at least weekly, but preferably during and after each clinical session. Consider appropriate time for feedback to be given possibly in dedicated slots.
3	In recognition of a reluctance to approach clinical teachers, the development of student literacy in the process should be addressed and prioritised. Students would like feedback should be both oral and written forms and given also for the excellent and good grades. It should have sufficient detail.
4	To address the manageability of feedback practices some consideration should be given to involving an element student self-assessment in the senior clinical years to promote their responsibility for recognising and achieving learning objectives in CLE. Students need to understand their part in the feedback process.
5	Consideration should be given to peer-to-peer discussion of cases lead by clinical teachers. Consider mentoring and communication training for CS. Useful to discuss prior to session what the student hopes to take away from the session (i.e., individualised learning outcomes on clinical sessions)

### Limitations and future directions

There are several limitations acknowledged in this study. This study is limited to one dental school experience and presents findings from a relatively small sample size which could reduce the generalisability of the findings to dental education in other universities and third level education in other healthcare disciplines. The data was not triangulated with other sources (e.g., teachers’ perspectives) which may have shed additional perspectives on the introduction of the MOF. It is planned to include this in future cycles of review of the MOF. Methodologies using questionnaires are also liable to response bias, focus groups were also performed as part of the study in an effort to overcome this form of bias. A 20-minute focus group, with six to eight participants in each group, could be considered somewhat short but we were able to gather sufficient feedback on the MOF in this time for the particular research questions. In further studies a greater duration for focus groups will be considered.

Perhaps, in this study, the once-off training delivered was insufficient for some clinical teachers in order to improve the feedback relationship between students and teachers which is at the centre of successful feedback practices and further mentoring and communication training is required. A further limitation is the lack of a comparison or control group in this study. Because a new MOF was introduced for all students, it is difficult to quantify this MOF versus the previous ways feedback was given or an alternative MOF. The student literacy in feedback practices is another area for exploration and may need to be addressed in future revisions of this study.

 Based on this evaluation, it is planned to incorporate the recommendations for how to improve the MOF into a revised version which will be subsequently evaluated, thereby creating an iterative cycle of review and improvement that responds agilely to the needs of teachers and students.

## Conclusions

The clinical environment is dynamic, stimulating and challenging; nevertheless, a high level of supervisor student dialogue and interaction is necessary to promote a safe and effective learning environment. With time constraints and high student to clinical teacher ratios there can be a tendency for learning to be reduced to purely observation and supervision which may be detrimental to learning. Feedback is at the heart of all learning, and it remains a complex and challenging process, but by adopting some approaches of effective and evidence-based feedback practice students can be supported in regulating their own learning and clinical teachers can be supported in delivering quality feedback. The findings from this study demonstrate that the introduction of a MOF, based on evidence-based principles and practices, can promote effective feedback process and address many challenges identified in the literature. This study outlines and recommends adaptations to the current MOF to help ensure quality feedback in the clinical learning environment.

## Supplementary Information

**Additional file 1.**

## Data Availability

The authors declare that the data supporting the findings of this study are available withing the article and the supplementary information files.
