# Supplementary material for: The evaluation of an evidence-based model of feedback implemented on an undergraduate dental clinical learning environment
Source: BMC Med Educ. 2022 Aug 1;22:588. doi: 10.1186/s12909-022-03630-1 (PMC9341118; doi:10.1186/s12909-022-03630-1)
Supplement: Supplementary file 1 — Additional file 1. [file 12909_2022_3630_MOESM1_ESM.docx]

## Supplemental Section.

Results and graphs 2

Appendix 1 16

Appendix 2 20

## Results and Graphs


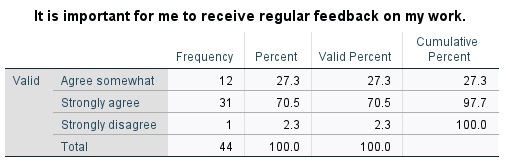


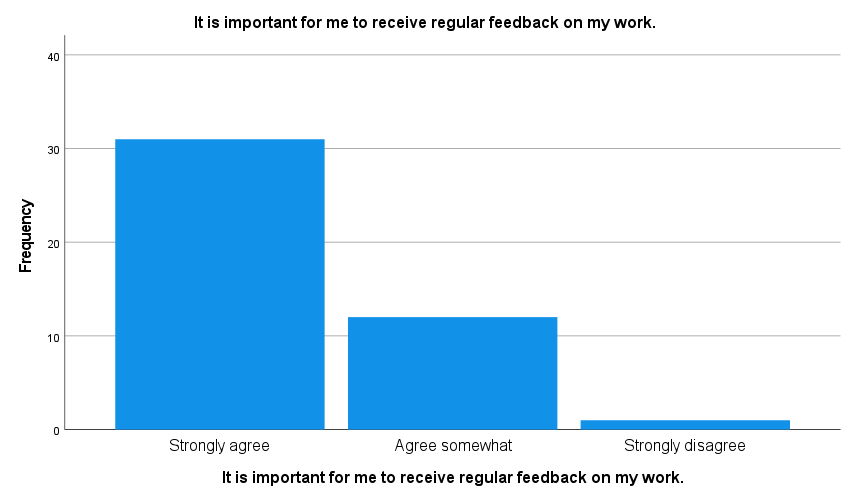


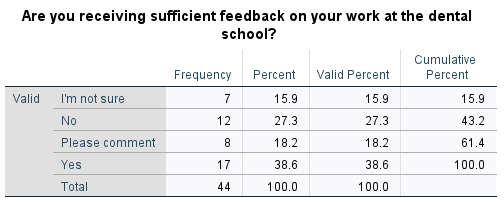


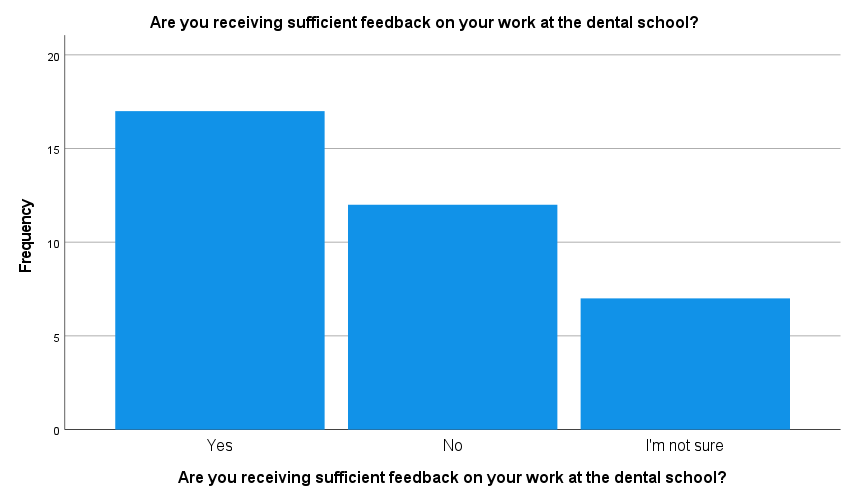


| **Are learning outcomes highlighted at the beginning of each term** | | | | | |
| --- | --- | --- | --- | --- | --- |
|  | | Frequency | Percent | Valid Percent | Cumulative Percent |
| Valid |  | 1 | 2.3 | 2.3 | 2.3 |
|  | I'm not sure | 4 | 9.1 | 9.1 | 11.4 |
|  | No | 15 | 34.1 | 34.1 | 45.5 |
|  | Please comment | 8 | 18.2 | 18.2 | 63.6 |
|  | Yes | 16 | 36.4 | 36.4 | 100.0 |
|  | Total | 44 | 100.0 | 100.0 |  |


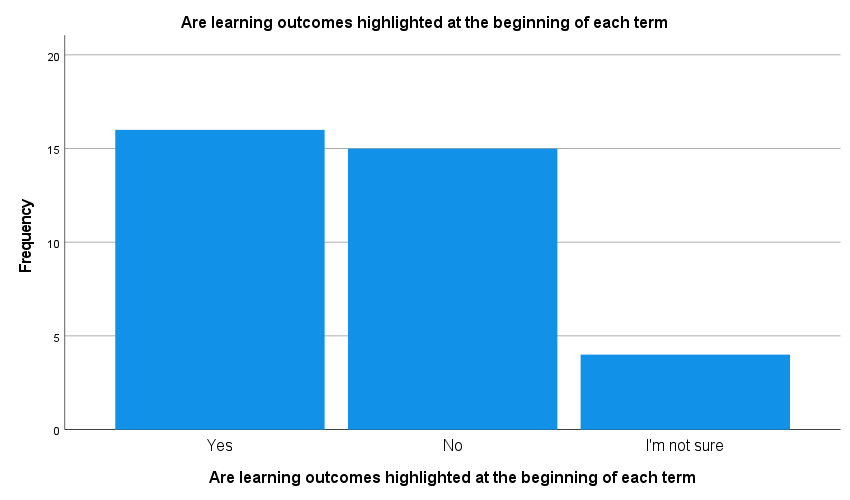


| **Are the learning outcomes outlined for each clinical session** | | | | | |
| --- | --- | --- | --- | --- | --- |
|  | | Frequency | Percent | Valid Percent | Cumulative Percent |
| Valid | I'm not sure | 3 | 6.8 | 6.8 | 6.8 |
|  | No | 19 | 43.2 | 43.2 | 50.0 |
|  | Please comment | 4 | 9.1 | 9.1 | 59.1 |
|  | Yes | 18 | 40.9 | 40.9 | 100.0 |
|  | Total | 44 | 100.0 | 100.0 |  |


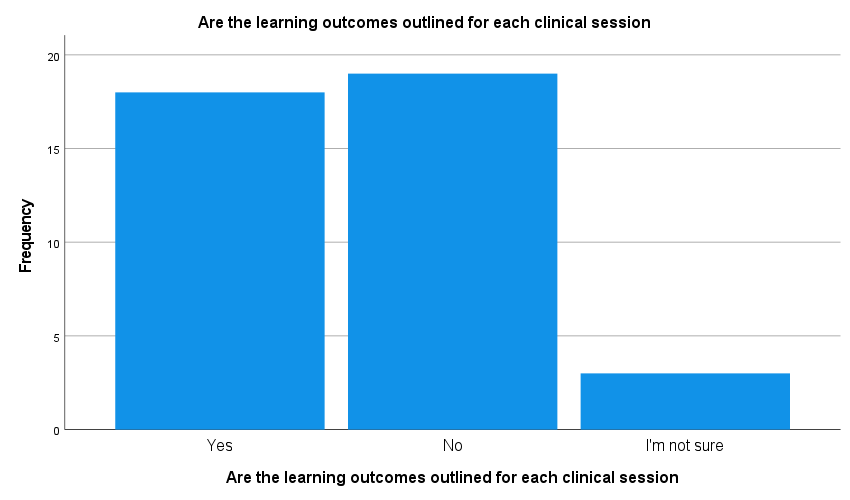


| **Does the feedback you receive enhance your performance?** | | | | | |
| --- | --- | --- | --- | --- | --- |
|  | | Frequency | Percent | Valid Percent | Cumulative Percent |
| Valid | I'm not sure | 3 | 6.8 | 6.8 | 6.8 |
|  | No | 2 | 4.5 | 4.5 | 11.4 |
|  | Other (please specify) | 5 | 11.4 | 11.4 | 22.7 |
|  | Yes | 34 | 77.3 | 77.3 | 100.0 |
|  | Total | 44 | 100.0 | 100.0 |  |


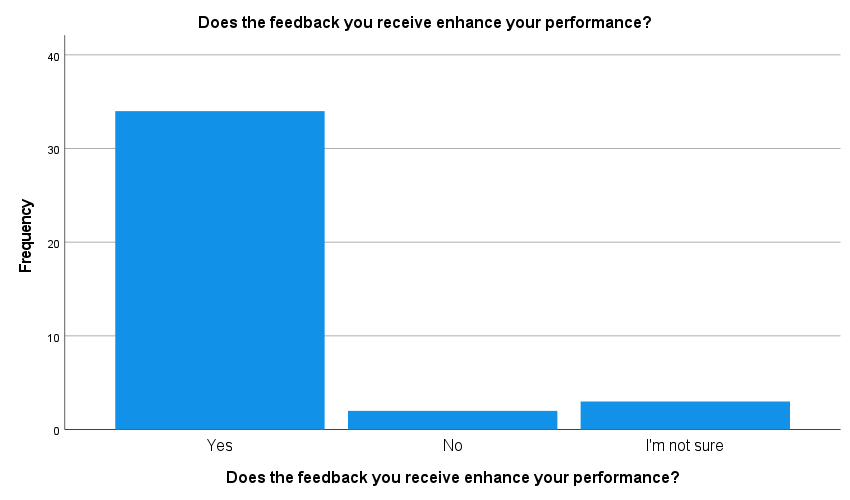


| **Does the feedback you receive in the session feed forward into what you can achieve the following week?** | | | | | |
| --- | --- | --- | --- | --- | --- |
|  | | Frequency | Percent | Valid Percent | Cumulative Percent |
| Valid |  | 1 | 2.3 | 2.3 | 2.3 |
|  | I'm not sure | 4 | 9.1 | 9.1 | 11.4 |
|  | No | 3 | 6.8 | 6.8 | 18.2 |
|  | Please comment | 2 | 4.5 | 4.5 | 22.7 |
|  | Yes | 34 | 77.3 | 77.3 | 100.0 |
|  | Total | 44 | 100.0 | 100.0 |  |


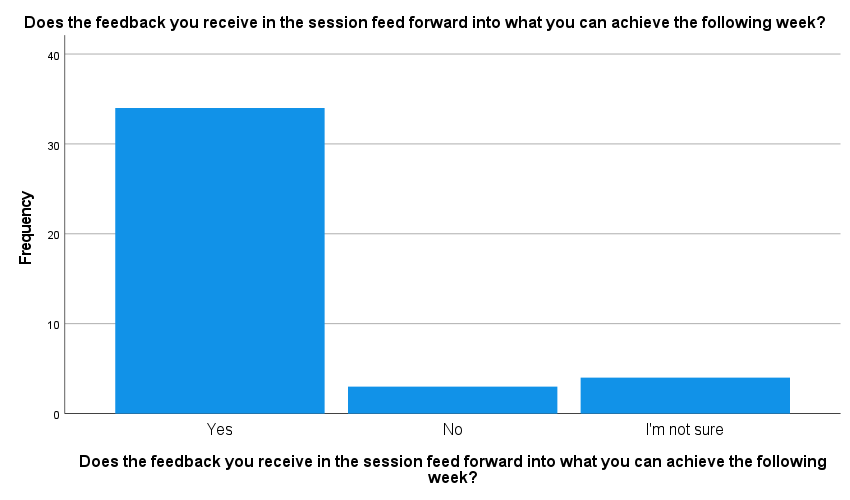


**What aspects of feedback do you value? Please choose all that apply**

|  | N | % |
| --- | --- | --- |
| I like to know where I went wrong | 37 | 84.1 |
| I like to know how I can improve on my work/grade | 38 | 86.4 |
| I feel feedback from a supervisor who is familiar with my work is more useful | 32 | 72.7 |
| I do not value feedback | 1 | 2.3 |

**Do any of the following issues limit your ability to get the feedback you need? Please choose all that may be applicable**

|  | N | % |
| --- | --- | --- |
| Time constraints | 29 | 65.9 |
| I find it difficult to engage with some of the supervisors/academic staff | 16 | 36.4 |
| I am able to assess my own work | 4 | 9.1 |
| The environment is not conducive to feedback seeking practices | 13 | 29.5 |

**How often do you like to receive feedback? (Tick all that apply)**

|  | N | % |
| --- | --- | --- |
| During and after every clinical session | 27 | 61.4 |
| Weekly | 10 | 22.7 |
| Every two weeks | 2 | 4.5 |
| Monthly | 5 | 11.4 |
| Twice a term | 1 | 2.3 |
| Once a term | 4 | 9.1 |

| **Do you feel able to discuss the feedback that you currently are receiving with your supervisors?** | | | | | |
| --- | --- | --- | --- | --- | --- |
|  | | Frequency | Percent | Valid Percent | Cumulative Percent |
| Valid | Don't know (please comment) | 4 | 9.1 | 9.1 | 9.1 |
|  | No | 13 | 29.5 | 29.5 | 38.6 |
|  | Yes | 27 | 61.4 | 61.4 | 100.0 |
|  | Total | 44 | 100.0 | 100.0 |  |


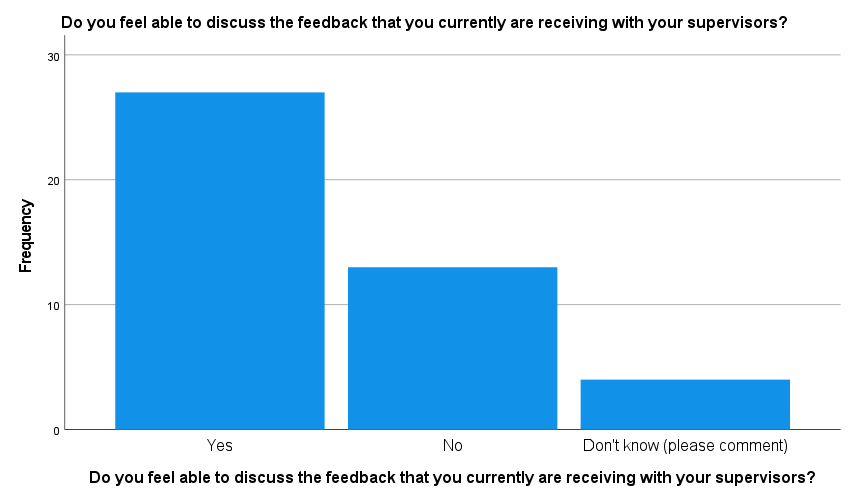


| **How would you prefer to receive feedback?** | | | | | |
| --- | --- | --- | --- | --- | --- |
|  | | Frequency | Percent | Valid Percent | Cumulative Percent |
| Valid | Oral | 15 | 34.1 | 34.1 | 34.1 |
|  | Written | 2 | 4.5 | 4.5 | 38.6 |
|  | Written and oral | 27 | 61.4 | 61.4 | 100.0 |
|  | Total | 44 | 100.0 | 100.0 |  |


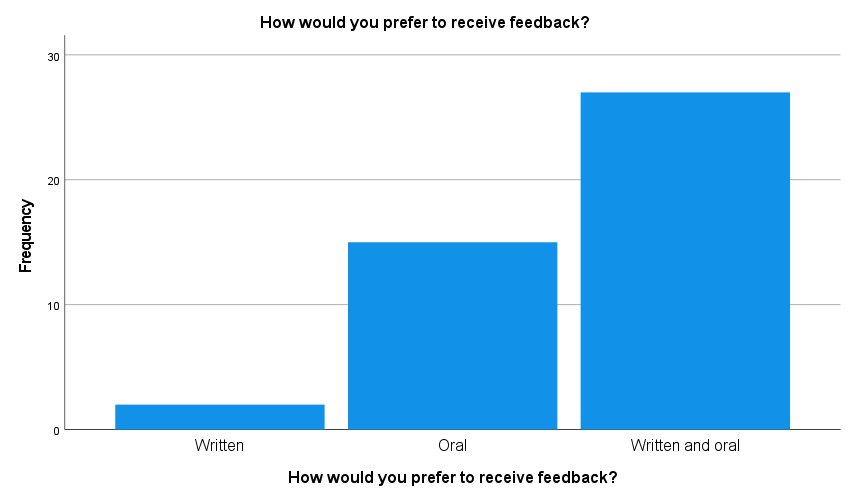


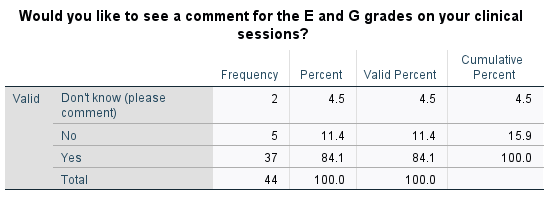


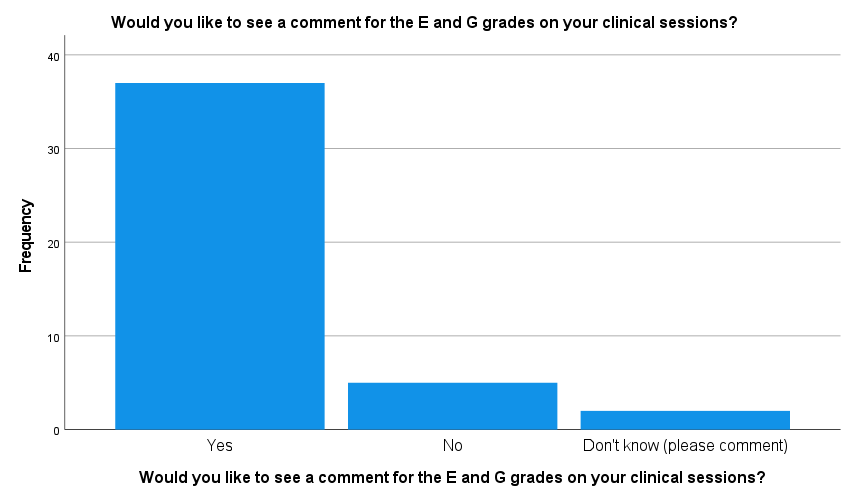


| **What do you think of the feedback processes at the dental school?** | | | | | |
| --- | --- | --- | --- | --- | --- |
|  | | Frequency | Percent | Valid Percent | Cumulative Percent |
| Valid |  | 1 | 2.3 | 2.3 | 2.3 |
|  | Good | 16 | 36.4 | 36.4 | 38.6 |
|  | Just satisfactory | 18 | 40.9 | 40.9 | 79.5 |
|  | Unsatisfactory | 9 | 20.5 | 20.5 | 100.0 |
|  | Total | 44 | 100.0 | 100.0 |  |


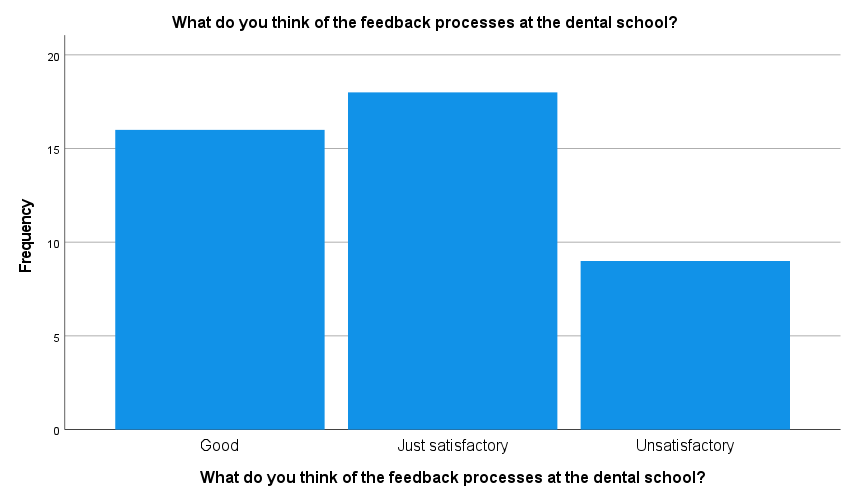


| **Would you like a change in the feedback you receive on the clinical sessions?** | | | | | |
| --- | --- | --- | --- | --- | --- |
|  | | Frequency | Percent | Valid Percent | Cumulative Percent |
| Valid | No | 6 | 13.6 | 13.6 | 13.6 |
|  | Not sure (please comment) | 8 | 18.2 | 18.2 | 31.8 |
|  | Yes | 30 | 68.2 | 68.2 | 100.0 |
|  | Total | 44 | 100.0 | 100.0 |  |


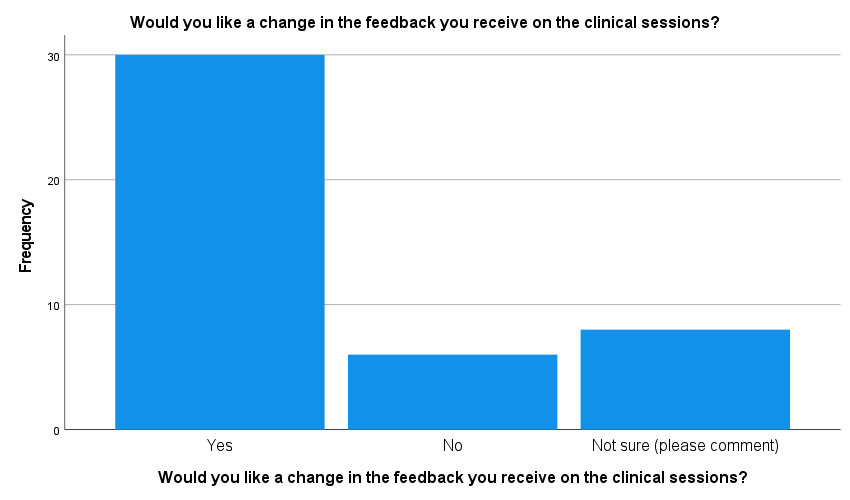


## Appendix 1. Anonymous online survey: evaluation of model of feedback.

**1. It is important for me to receive regular feedback on my work.**

Strongly agree

Agree somewhat

Uncertain

Disagree

Strongly disagree

**2. Are you receiving sufficient feedback on your work at the dental school?**

Yes

No

I'm not sure

Please comment

**3. Are learning outcomes highlighted at the beginning of each term**

Yes

No

I'm not sure

Please comment

**4. Are the learning outcomes outlined for each clinical session**

Yes

No

I'm not sure

Please comment

**5. Does the feedback you receive enhance your performance?**

Yes

No

I'm not sure

Other (please specify)

**6. Does the feedback you receive in the session feed forward into what you can achieve the following week?**

Yes

No

I'm not sure

Please comment.

**7. What aspects of feedback do you value? Please choose all that apply**

I like to know where I went wrong

I like to know how I can improve on my work/grade

I feel feedback from a supervisor who is familiar with my work is more useful

I do not value feedback

Please comment.

**8. Do any of the following issues limit your ability to get the feedback you need? Please choose all that may be applicable**

Time constraints

I find it difficult to engage with some of the supervisors/academic staff

I am able to assess my own work

The environment is not conducive to feedback seeking practices

Please comment.

**9. How often do you like to receive feedback?**

During and after every clinical session

Weekly

Every two weeks

Monthly

Twice a term

Once a term

**10. Do you feel able to discuss the feedback that you currently are receiving with your supervisors?**

Yes

No

Don't know (please comment)

Other (please specify)

**11. How would you prefer to receive feedback?**

Written

Oral

Written and oral

Please comment

**12. Would you like to see a comment for the E and G grades on your clinical sessions?**

Yes

No

Don't know (please comment)

Please comment

**13. What do you think of the feedback processes at the dental school?**

Very good

Good

Just satisfactory

Unsatisfactory

Comment

**14. Do you think the model of feedback was a good intervention on the clinical sessions?**

Yes

No

Not sure (please comment

**15. Was it successful in your opinion?**

Yes

No

Not sure (please comment)

**16. Was there any point in the model of feedback which you had difficulty with?**

Yes

No

Not sure (please comment)

**17. Would you like to continue using this model of feedback on clinical sessions?**

Yes

No

Not sure (please comment)

## Appendix two: Focus group questions

Aim of the focus group was to evaluate if the students found the model of feedback enhanced their learning

Questions to guide the focus group (15 mins approx.)

**I Evaluation of the use of the model of feedback weeks 4-8.**

1. What are your thoughts on the model of feedback implemented weeks 4-8?

2. Would you say you were satisfied with this model?

3. If so, what is going well, why is that?

4. If not what didn’t go so well, why is that?

5. How about the aspect of reflection highlighted in the model? What do you think of that?

6. Are there things you are dissatisfied with feedback that you want changed?

7. Do you think this is a valuable tool and something you would like to see continued?

8. Did your supervisors use the model? Were there any issues with this?

9. Are there other recommendation or suggestions you would like to make?

**II Beliefs about feedback**

1. What does good feedback look like to you?

2. Do you have a role in the feedback process? If so, what is it?

3. Do you ever actively seek out feedback, when and why?

4. What specific feedback needs do you think you have and why?

5. Do you ever have to give feedback to others?

6. If yes, to question 5, does giving feedback to others enhance you own understanding of the work at all? If so how and why? Has feedback helped you to make progress? Give examples.

7. Are you able to self-assess your own standard of work? Explain your answer

**III Type of feedback given and its appropriateness for your learning.**

1. Who gives you feedback on your work?

2. Whose feedback matters most to you and why? (Lecturer, peer, family friends?)

3. Which one mode of feedback or combination of feedback (written, email, text, audio, is most valuable to you and why?

4. What form of feedback (oral v written; written v typed) is most valuable and why
